# Functional Modules, Structural Topology, and Optimal Activity in Metabolic Networks

**DOI:** 10.1371/journal.pcbi.1002720

**Published:** 2012-10-11

**Authors:** Osbaldo Resendis-Antonio, Magdalena Hernández, Yolanda Mora, Sergio Encarnación

**Affiliations:** Centro de Ciencias Genómicas-UNAM, Col. Chamilpa, Cuernavaca, Morelos, México; Stanford University, United States of America

## Abstract

Modular organization in biological networks has been suggested as a natural mechanism by which a cell coordinates its metabolic strategies for evolving and responding to environmental perturbations. To understand how this occurs, there is a need for developing computational schemes that contribute to integration of genomic-scale information and assist investigators in formulating biological hypotheses in a quantitative and systematic fashion. In this work, we combined metabolome data and constraint-based modeling to elucidate the relationships among structural modules, functional organization, and the optimal metabolic phenotype of *Rhizobium etli*, a bacterium that fixes nitrogen in symbiosis with *Phaseolus vulgaris*. To experimentally characterize the metabolic phenotype of this microorganism, we obtained the metabolic profile of 220 metabolites at two physiological stages: under free-living conditions, and during nitrogen fixation with *P. vulgaris*. By integrating these data into a constraint-based model, we built a refined computational platform with the capability to survey the metabolic activity underlying nitrogen fixation in *R. etli*. Topological analysis of the metabolic reconstruction led us to identify modular structures with functional activities. Consistent with modular activity in metabolism, we found that most of the metabolites experimentally detected in each module simultaneously increased their relative abundances during nitrogen fixation. In this work, we explore the relationships among topology, biological function, and optimal activity in the metabolism of *R. etli* through an integrative analysis based on modeling and metabolome data. Our findings suggest that the metabolic activity during nitrogen fixation is supported by interacting structural modules that correlate with three functional classifications: nucleic acids, peptides, and lipids. More fundamentally, we supply evidence that such modular organization during functional nitrogen fixation is a robust property under different environmental conditions.

## Author Summary

Biological networks are an inherent concept in systems biology that is useful in elucidating how biological entities—as metabolites or proteins—work together in supporting specific phenotypes in microorganisms. Notably, topological analyses carried out over these networks have shown that modular organization is a ubiquitous property at different levels of biological organization, in such a way that modular organization may serve as an organizing principle governing the metabolic activity in microorganisms. With the aim of elucidating the relationship among functional modules, network topology, and optimal metabolic activity, here we present an integrative study that combines computational modeling and metabolome data for evaluation of the metabolic activity of the soil bacterium *Rhizobium etli* during symbiotic nitrogen fixation with *Phaseolus vulgaris*. As a result, we supply experimental and computational evidence supporting the concept that the optimal metabolic activity during this biological process is guided by modular structures in the metabolic network of *R. etli*. Even more fundamentally, we suggest that these biochemical modules interact among each other to ensure an optimal phenotype during nitrogen fixation. Finally, through the *in silico* analysis on the genome scale metabolic reconstruction for *R.etli*, we give some examples that suggest that these modular structures supporting nitrogen fixation are robust to external physiological conditions.

## Introduction

With the advent of bioinformatics and high-throughput technologies, plentiful sources of information stored in databases are now available to help unveil how a variety of biological entities interact among each other and to elucidate how these interacting networks support phenotypic behaviors in microorganisms. Notably, a variety of studies accomplished with these networks have contributed to elucidation of some fundamental organizing principles by which the cell presumably regulates and coordinates its vital biological functions. Among these organizing properties, structural modularity is a systemic property that has been observed in a variety of biological networks, which range from genetic transcriptional regulation to metabolic activity [1], [2], [3], [4], [5]. On an even more fundamental level, there is evidence that these modules inferred from network topology can be associated with functional modules; these latter are defined as a set of biological components (metabolites, proteins, or genes) that coordinately participate in accomplishing a specific biological function in the cell [6].

On the other hand, the idea that optimization principles guide metabolic activity in microorganisms has been an interesting hypothesis that, in combination with genome-scale metabolic reconstructions, has resulted in a successful framework for a systematic, quantitative, and predictive scheme in systems biology [7]. Briefly, the optimization problem in this context is reduced to identification of the metabolic flux along the network that ensures maximal production of a specific array of metabolites representing a specific phenotypic state in the microorganism. An optimal metabolic phenotype constitutes the basis of constraint-based modeling, and its application domain has been extended to a variety of organisms in the past few decades [8], [9].

In this context, an immediate and fundamental question emerges: how do these structural modules organize and coordinate among themselves to support an optimal metabolic phenotype in microorganisms?. Even though this enterprise is far from being solved, some remarkable advances have been reported in the field. For instance, a recent experimental and *in silico* study on a metabolic reconstruction for *Escherichia coli* supported the idea that feedback inhibition in metabolic units, called modules, constitutes a mechanism capable of inducing an optimal growth rate [10]. Equally relevant, there are studies that have pointed out that modular organization on a genomic scale may be a natural strategy for coordinating the transcriptional and metabolic activities required for ensuring that cells evolve and efficiently respond to environmental perturbations [1], [5]. Despite these and other advances, the study of the principles governing the metabolic organization in cells is in its infancy, and additional discoveries are required for surveying how structural modules in a metabolic network link together to efficiently achieve their biological functions [10].

In this work, by using a systems biology description, we supply computational and experimental evidence that suggests that structural and functional modularities are robust properties when the cell operates under its optimal metabolic phenotype at different physiological conditions. To support our conclusions, we carried out an integrative study involving computational modeling and high-throughput sequencing technology for characterizing the metabolic activity of *Rhizobium etli* CFN42 during symbiotic nitrogen fixation in symbiotic association with *Phaseolus vulgaris* (bean plant). Here, this organism is our benchmark model, a decision that was favored based on the availability of 1) a computational description of its genome-scale metabolic reconstruction, 2) an integrative description among high-throughput technologies at nitrogen fixation stages, and 3) the valuable physiological knowledge already available that describes the metabolic activity during nitrogen fixation by this organism in symbiotic association with *P. vulgaris* [11].

Hence, to elucidate the metabolic activity of *R.etli* during nitrogen fixation, constraint-based modeling was applied on an updated version of the metabolic reconstruction for this organism (*iOR450*) [11]. At present, the metabolic reconstruction of *R. etli* is an integrated network of 402 reactions involving the participation of 450 genes and 377 metabolites. Unlike our previous studies, here we used metabolome technology to experimentally support our *in silico* interpretations of *R.etli* metabolism under two physiological conditions: when it fixes nitrogen in symbiosis with *P. vulgaris*, and under free-living conditions with succinate and ammonium as carbon and nitrogen sources, respectively (see the Materials and Methods section).Thus, by applying capillary electrophoresis and mass spectrometry (CE-MS) [12], [13] to *R.etli* and its products, we report the relative abundances of 220 metabolites under these physiological conditions. To improve the interpretations obtained from the constraint-based modeling, we used metabolome data to identify metabolites with meaningful biological roles in bacterial nitrogen fixation. Consequently, this information was used to guide the reconstruction of a more proper objective function (OF) to computationally simulate this biological process. Next, with the results of our integrative analysis carried out between the constraint-based modeling and the metabolome data for *R. etli*, we concluded that the metabolic activity at optimal nitrogen fixation is supported by structural modules with well-defined functional activities. Furthermore, our *in silico* study let us show that those modular structures tend to be robust during changes in environmental conditions. Overall, our study supplies evidence that modular organization in metabolic networks is required for promoting an optimal metabolic phenotype in microorganisms.

## Results

### Metabolome profile in bacterial nitrogen fixation

**Figure 1:**
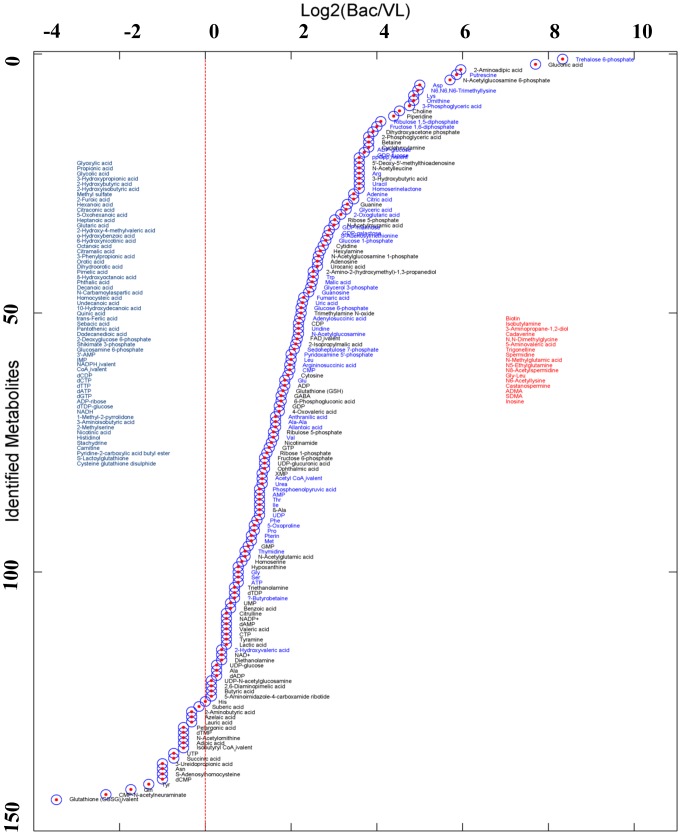
Experimental metabolome profile. Log-ratios of 220 metabolites detected under two physiological conditions for *R. etli*: bacteroids during nitrogen fixation (Bac) and free-living conditions (VL). Meanwhile, metabolites (shown in marine blue at the left side of the illustration) detected exclusively under free-living conditions are listed, and the metabolites denoted in red were exclusively detected during bacterial nitrogen fixation (listed on the right side). The rest of the metabolites were detected under both physiological conditions at different ratios (see the middle part of the figure).

In addition to transcriptome and proteome approaches, metabolome technology represents a third complementary approach to characterize the phenotypic state of a microorganism, through quantitative and qualitative descriptions of its metabolite concentrations. In order to elucidate the metabolic capabilities and organizing properties of *R. etli* metabolism, we investigated the metabolome profiles of this bacterium in two physiological situations: during nitrogen fixation with *P.vulgaris* and under free-living conditions (see the Materials and Methods section and reference [11]). Biological samples from each physiological condition were analyzed in triplicate using CE-MS technologies [12], [13], and the output was used to sense the relative abundance levels of metabolites under each physiological condition. Metabolome measurements were carried out through a facility service at Human Metabolome Technology Inc., Tsuruoka, Japan. Briefly, the samples were prepared following an experimental protocol supplied by Human Metabolome Technology Inc., and the ionic metabolites were separated through electric fields. Having separated the ionic metabolites by capillary electrophoresis the samples were subjected to spectrometric analysis, and their identities were selectively detected by monitoring ions over a large range of *m*/*z* values. Thus, spectrum profiles were compared in consultation with the Human Metabolome Technology database and normalized with respect to internal controls for uncovering and estimating their identities and metabolic abundances in both physiological conditions. The relative peak area observed by spectrometry and the average, standard deviation, and log-ratio obtained for each metabolite are reported in Dataset S1 in the supporting information and visually depicted in Figure 1. Overall, high-throughput analysis led us to identify and characterize the abundances of 220 ionic metabolites (93 cations and 127 anions) under both physiological conditions. A biological analysis of these results led us to arrive at the following conclusions:

1. A previous analysis based on transcriptome and proteome data led us to identify certain proteins required for the synthesis of different amino acids in *R. etli* during nitrogen fixation (arginine, tyrosine, tryptophan, phenylalanine, and lysine) [11]. In parallel with these findings, our current metabolome analysis led us to detect all the common amino acids, with the exception of cysteine; in this case we only identified its homologue, homocysteine. In light of these results, the participation of a variety of amino acids in *R. etli* during symbiotic nitrogen fixation with *P. vulgaris* is clear. The role of each amino acid and which of them are essential to carry out the biological processes are some avenues to be explored in the future.
2. With dicarboxylic acids as the carbon source, the TCA cycle would be expected to be active. As we reported previously, an integrative analysis using high-throughput technology (on the transcriptome and proteome levels) and constraint-based modeling suggested that the TCA cycle is active during nitrogen fixation [11]. Consistent with this finding, five metabolites that participate in the TCA cycle were detected from *R. etli* (citric acid, 2-oxoglutaric acid, succinic acid, fumaric and malic acid). In our opinion, our new high-throughput data supply additional evidence that an operational TCA cycle supports bacterial nitrogen fixation.
3. Purine and pyrimidine pathways are important during nodulation processes, given that most purine or pyrimidine auxotrophs in *Rhizobiaceas* are ineffective in symbiotic nitrogen fixation because they elicit pseudo-nodules that are devoid of an infection thread [14].In parallel, our earlier results indicated that an intense synthesis of macromolecules, such as purines and pyrimidines, is carried out in bacteria [11]. In support of these physiological descriptions and with an emphasis on the importance of these molecules during the nitrogen fixation process, we identified the purines—GTP, guanine, adenosine, guanosine, ADP, GDP, allantoic acid, XMP, AMP, GMP, ATP, dAMP, dADP, dAMP,and dADP—and the pyrimidines—CDP,uridine, CMP, cytosine, UDP, UMP,dTMP,UTP,dTDP, CTP, and cytidine—through our metabolome study.
4. In a previous report, we suggested that besides gluconeogenesis, a fueling pathway based on pentoses may exist in bacteria during nitrogen fixation [11].In agreement with this hypothesis, ribose-5-phosphate, fructose-1,6-diphosphate, ribulose-1,5-diphosphate, ribulose-5-phosphate, ribose-1-phosphate, sedoheptulose-7-phosphate, and fructose-6-phosphate metabolites were detected in the bacteroids. In fast-growing rhizobia, the pentose phosphate pathway in combination with the Entner-Doudoroff pathway are probably the major routes used for the metabolism of sugars [15]. Thus, based on this observation, we hypothesize that other carbon sources, in addition to dicarboxylic acids, participate in bacterial nitrogen fixation. This hypothesis constitutes a perspective that should be experimentally verified in future works.

Undoubtedly, the above descriptions are valuable for elucidation of the metabolic landscape during bacterial nitrogen fixation in *R.etli*; however, in order to explore the relationships among network topology, functionality, and optimal metabolic phenotype, it is necessary to move toward an integrative, quantitative, and predictive description. To this end, metabolome data were used for improving and assessing the flux metabolic activity inferred from constraint-based modeling when applied on the genome-scale metabolic reconstruction of *R. etli*. Thus, from the set of 220 metabolites experimentally detected, we identified 119 of the 377 metabolites that participate in the metabolic reconstruction reported for *R. etli OR450* [11]. This metabolic set covered 32.75% of the total number of metabolites participating in the metabolic reconstruction and constituted our experimental dataset for improving, supporting, and assessing the results that emerged from the constraint-based modeling.

### Bridging metabolome data and constraint-based modeling results

Constraint-based modeling is a paradigm in systems biology for exploring the metabolic phenotype in cells under specific environmental conditions and/or subject to genetic perturbations [16], [17], [18]. In particular, Flux Balance Analysis (FBA) has proven to be a useful computational tool for surveying the metabolic phenotype capacity in a microorganism and to simultaneously evaluate its coherent description with high-throughput technology [9], [11]. Briefly, once the genome-scale metabolic reconstruction for an organism has been completed, FBA can be divided into two main steps: 1) the reconstruction of an OF that simulates a particular phenotypic state for the microorganism (for instance, the growth rate or maximal nitrogen fixation, to name two), and 2) the search for a flux distribution along the entire network that maximizes the selected OF when the system is at steady-state [7]. Among the variety of applications that can be tackled with this systems-level framework, bacterial nitrogen fixation carried out by *R.etli* is an example of how an integrative study using computational modeling and high-throughput technology can contribute to understanding, predicting, and elucidating the metabolic activity during this biological process [11]. Based on these previous achievements, we selected this organism as our model system for surveying the relationships among structural organization, functionality, and the maximal phenotype during bacterial nitrogen fixation.

Before proceeding with our *in silico* analysis, we took advantage of the metabolome data to refine and improve a previous FBA carried out for this organism [11]. Thus, given the metabolic reconstruction *iOR450*, we improved the FBA by suggesting a more proper OF, one capable of modeling bacterial nitrogen fixation in a more accurate and realistic fashion. Reconstruction of an OF is a crucial issue in constraint-based modeling for computationally mimicking the metabolic activity of a microorganism, in this case, bacterial nitrogen fixation. For this reason, an OF is mainly defined in terms of those metabolites whose permanent production is essential for sustaining bacterial nitrogen fixation [11], [19]. Currently, the OF for modeling bacterial nitrogen fixation has been entirely constructed in terms of a query in the scientific literature [11], [19]; however, the metabolome data depicted in Figure 1 open the possibility for identifying new potential metabolites that were not included in previous analyses due to a lack of physiological evidence.

The issue described above is extremely important because it directly influences the quality of the *in silico* interpretations and the coherent description based on the combination with high-throughput data. In order to proceed with this improvement, our strategy was based on the argument that metabolites detected at higher concentrations at the symbiotic nitrogen fixation stage, compared with those observed under free-living conditions, may mirror their fundamental participation in sustaining a functional phenotype during the biological process. Hence, under this assumption, we identified those metabolites whose concentrations increased during nitrogen-fixing activity by applying a statistical test on the log-ratio data, as shown in Figure 1 and Dataset S1 in the supporting information. To reduce the probability of including false-positive results, we limited the selection of metabolites to only those that obeyed the following conditions: 1)the log-ratio increased by at least by 2-fold during nitrogen fixation, with a *p*-value<0.01 (see the Materials and Methods section); and 2)once the candidate metabolite was included in the OF, there existed at least a solution for the metabolic flux distribution that maximized the OF at steady state.

**Figure 2:**
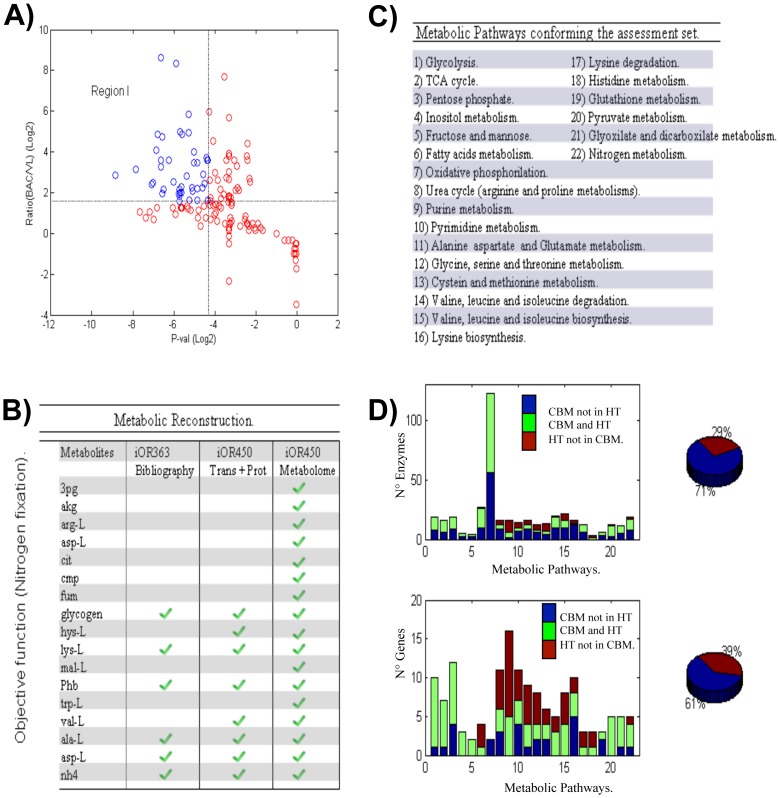
Integrative description among *in silico* analysis and high-throughput data. (A) Log-ratio and *p*-values plot. In blue we show those metabolites that obeyed two of three criteria: a log-ratio higher than 2, a *p*-value lower than 0.01,and its requirement for consideration as a metabolite with an important role in supporting bacterial nitrogen fixation. (B) Historical improvements in OFs. Since the first publication dealing with the systems biology description of the bacterial nitrogen fixation for *R. etli* [19], some improvements have been carried out for determining the OF, by identifying proper metabolites required for supporting this biological process (see Figure 2). At present, the suggested objective function is the result of a careful search of the published literature and an integrative description in the metabolome data. Under these experimental and physiological bases, our modeling located an OF slightly closer to the complex metabolism supporting bacterial nitrogen fixation, and consequently constraint-based modeling raised its accuracy and predictive scope. D) Comparative analysis between FBA and transcriptome and proteome data. A detailed comparison between computational predictions and high-throughput data was carried out for all 22 metabolic pathways defined in panel C. One can distinguish three possible situations: 1) genes (enzymes) that were predicted *in silico* but not monitored experimentally (CBM not HT; blue); 2) genes (enzymes) that were consistently observed in both schemes (CBM and HT; green); and 3) genes (enzymes) that were experimentally monitored but not predicted *in silico* (HT not CBM; red). As explained in the Materials and Methods section, *η* is related to the fraction of genes (enzymes) that were consistently observed in both schemes and constitutes the backbone for our modeling assessment. Upper and lower bars at the right side of panel D indicate the fraction of proteins and genes that were predicted *in silico* and detected in proteome or transcriptome data.

A graphical representation of those metabolites obeying the first criterion is shown in Figure 2 (A) (blue points). Consequently, by applying the second criterion over this metabolite subset, we identified nine metabolites that potentially have an important role in driving and supporting bacterial nitrogen fixation. Having identified candidate metabolites, we integrated them into the OF and applied linear optimization to identify the metabolic flux distribution that maximized the metabolism of nitrogen-fixing bacteria (see the Materials and Methods section). The time line for the metabolic components, integrating the previous OF in bacterial nitrogen fixation for *R. etli* (*Z^Fix^*),including the one obtained with the criteria described above, is summarized in Figure 2 (B) and the Materials and Methods section. As a result, the OF reported in this study shows a significant number of components that were not included in previous versions, and among them are the following: 3-phospho-d-glycerate (3pg),2-oxoglutarate (akg), l-arginine (arg-L), l-aspartate (asp-L), citrate (cit), CMP (cmp), fumarate (fum), malate (mal-L), and l-tryptophan (trp-L)[see Figure 2(B)].

To assess the consequences of this improvement and compare the results reported here with those obtained in our previous study, we performed FBA on the metabolic reconstruction for *R. etli* (*iOR450*), taking into account the new OF depicted in Figure 2(B). To proceed with this comparative analysis, our simulation was carried out under equivalent conditions as those described in reference [11], which can be summarized as follows:

1. The experimental data underlying this analysis were integrated on those genes up-regulated and the proteins detected during bacterial nitrogen fixation after 18 days of inoculation of *R. etli* with root plant of *P. vulgaris* (see the Materials and Methods section). Overall, this set of high-throughput data was integrated based on 415 proteins and 689 up-regulated genes (see the details in reference [11]).
2. In analogy with a previous analysis, we selected 22 KEGG metabolic pathways to evaluate the concordance between the metabolic activity predicted *in silico* and that interpreted with the high-throughput technology. Figure 2 (C) enlists that set of metabolic pathways. Even though the KEGG database reports around 300 different metabolic pathways for *R. etli*, we selected only 22 as the core of our analysis because transcriptome and proteome data available for this organism have made evident the participation of the 22 pathways during bacterial nitrogen fixation [11]. According to the KEGG database, these 22 metabolic pathways contain 652 genes for *R. etli*, of which around 54% were included in the metabolic reconstruction for *iOR450*. This set of genes and their corresponding enzymes constituted the central core for evaluating the coherence between *in silico* predictions and high-throughput data interpretations. Even though the *in silico* assessment relied on the activity of these 22 metabolic pathways, the FBA took into account all the reactions included in the entire metabolic reconstruction. We expect that this latter consideration is valuable for exploring and predicting the metabolic role that additional pathways may have on bacterial nitrogen fixation.
3. To quantify the concordance between the metabolic activity predicted *in silico* and that interpreted from high-throughput technology, we calculated the fraction of genes (*η^Genes^*) and enzymes (*η^Enzymes^*)over the 22 pathways that were predicted to be active by constraint-based modeling and simultaneously detected in the high-throughput data (see the Materials and Methods section). The numerical values of these parameters, were defined in such a way that 1 represented the highest and 0 the lowest consistency score between the genes (or enzymes) predicted by both procedures.

In this context, FBA carried out for *iOR450* with the new OF led us to conclude that the consistency coefficients for genes (*η^Genes^*)and proteins (*η^Enzymes^*) were 0.61 and 0.71, respectively [see Figure 2 (D)]. Given that the new OF was based on the experimental metabolome profile, we argue that our *in silico* analysis moves towards a more accurate and improved description of metabolic activity during bacterial nitrogen fixation.

In order to evaluate the metabolic implications of these results and compare our results with previous reports carried out for bacterial nitrogen fixation by *R. etli*, we report in Table 1 the consistency coefficients and the degrees of coverage obtained in each case. Here, the degree of coverage is defined as the fraction of genes (proteins), from the total number of genes (proteins) obtained from constraint-based modeling, that were detected by transcriptome or proteome technology (see the data reported in reference [11]). As Table 1 shows, the values of the consistency coefficients for genes and proteins had slight variations in each case. However, as Table 1 chronologically shows, the OF has systematically improved two variables: the total number of genes (proteins) obtained from constraint-based modeling, and the subset of genes (proteins) belonging to this group detected by transcriptome or proteome technologies. Thus, the OF reconstructed in this work led us to increase the degree of coverage compared with previous cases: 72 of 101 enzymes predicted *in silico* were experimentally identified with high-throughput technologies. In agreement with this finding, 187 genes were experimentally identified by high-throughput technologies from the 306 genes predicted in the *in silico* analysis. In general terms, we conclude that while the consistency coefficients slightly varied among them, the OF constructed here induced the activity of additional reactions for the biosynthesis of new metabolites in bacterial nitrogen fixation. We consider that the latter issue is a crucial step in moving toward a more accurate description for characterizing and understanding the metabolic activity supporting bacterial nitrogen fixation and eventually uncovering their fundamental organizational principles at this biological level.

**Table 1:** Comparative analysis between *in silico* results and high-throughput data for the three OFs representing bacterial nitrogen fixation in *R. etli*.

	Year	Data for evaluating or constructing the OF	Consistency coefficient		Degree of coverage*		Reference
			Genes	Enzymes	Gene	Enzymes	
**Objective Function**	2007	Literature review	0.6	0.67	165/272	50/74	[16]
	2011	Transcriptome/Proteome	0.69	0.76	173/249	63/82	[5]
	2012	Metabolome	0.61	0.71	187/306	72/101	This study

* This parameter represents the fraction of genes (proteins) identified by constraint-based modeling that were experimentally detected by high-throughput technology.

### Structural modules and their functional compositions

The predominance of a hierarchical organization in a metabolic network seems to have advantages that can be observed on short-term and longer-term time scales, because structural modularity has been suggested as a fundamental network property by which cells evolve and orchestrate their physiological responses [3], [4], [20]. In order to elucidate the relation between structural modules and biological functionalities in the metabolic activity of *R. etli*, we focused first on identification of the structural modules in the genome-scale metabolic reconstruction for *iOR450* and, consecutively, analyzed the biological roles of their metabolic components.

**Figure 3:**
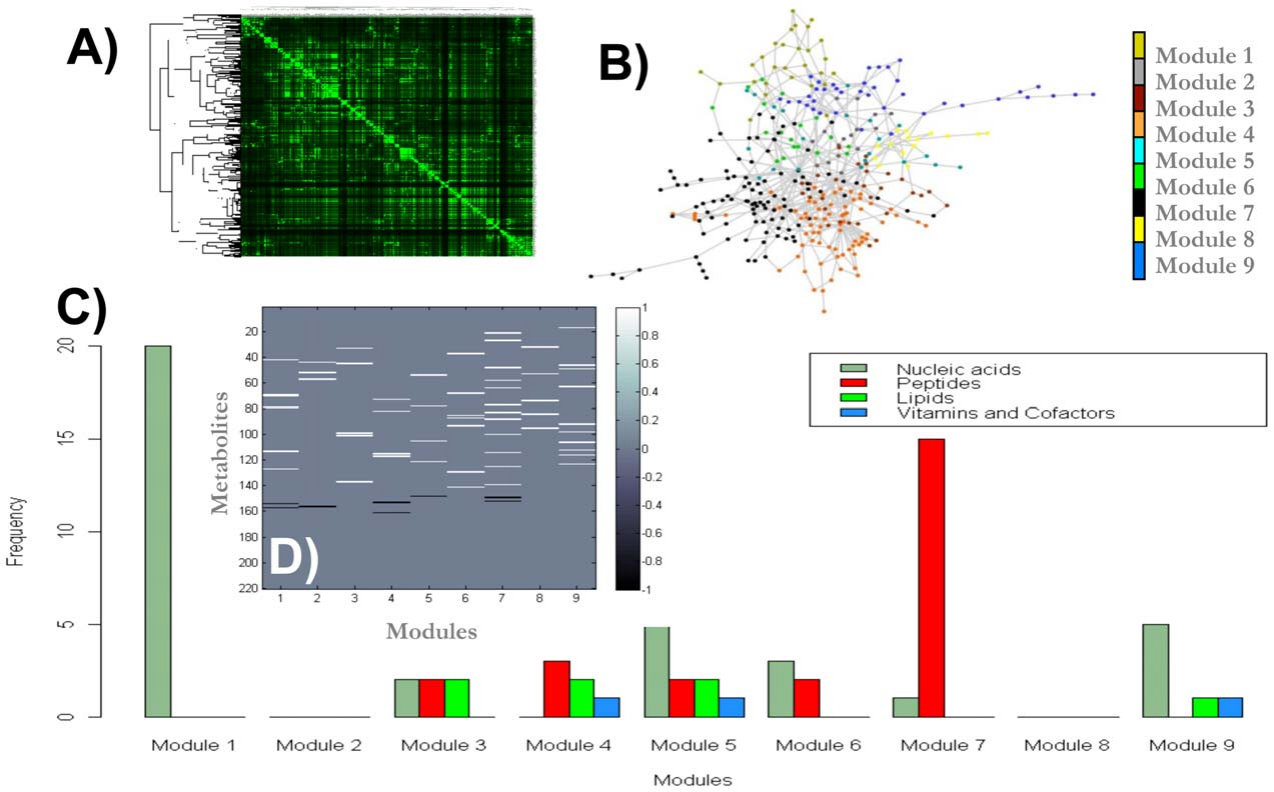
Modularity in the metabolic reconstruction for *R. etli*. The structural modules identified in the entire metabolic reconstruction are shown in panels (A) and (B).The color bar on the right indicates the different modules identified by their topological closeness in their components. The functional compositions of each structural module are shown in (C). In panel (D) we present the relative increment (white) or decrement (black) on concentration for those metabolites experimentally detected along the nine functional modules.

In terms of the first issue, modular structures were identified by considering a pure topological criterion, which has been suggested as a useful method for surveying the organization in a biological network [4], [5]. Hence, as we explained in the Materials and Methods section, we defined a metric of closeness for each pair of metabolites in the network by calculating the inverse square of the minimal path length between them. Consequently, by taking into account that the metabolites integrating a module are those whose numerical values of the metric tend to be similar, we classified those metabolites with a similar pattern of closeness through a hierarchical clustering analysis (see the Materials and Methods section). As a result of this analysis, nine topological modules were identified over the entire metabolic reconstruction [see Figure 3 (A) and (B)].

Even though the previous finding supplied information about the metabolic organization underlying the reconstruction, it was necessary to carry out a functional analysis to determine how these modules can be linked and support a specific metabolic phenotype. To this end, we proceeded to characterize the biological role of the set of metabolites that comprised each topological module defined in Figure 3(B). The functional composition of each module was determined by identifying the biological roles of their components, which in turn were defined according to the classification reported in the KEGG database [6]. By considering only those metabolites appearing in the KEGG database classification, we noted that the identified metabolites fell into three main biological groups: nucleic acids, peptides, and lipids [see Figure 3(C)]. This functional analysis suggested that most of the modules were formed by a heterogeneous composition among the three biological functionalities described above; however, we noted that two modules were characterized by a well-defined functional activity, *i.e.*, production of nucleic acids and peptide groups [see modules one and seven in Figure 3 (C)].

Intuitively, one can expect that the metabolites integrating a functional module can be characterized by their coordinate response to physiological changes in such a way that the relative concentrations of the metabolites comprising the modules be up or down-regulated in a coherent fashion under both physiological conditions. To qualitatively assess this assumption, we first identified those metabolites that were experimentally detected by high-throughput technology in each modular structure defined in Figure 3(B). Consequently, in order to estimate the coordinate behavior of metabolites inside modules, we evaluated their relative changes under the two physiological conditions: nitrogen fixation versus free-living conditions. Notably, most of the metabolites experimentally detected suggested that components inside modules up-regulate their concentrations during bacterial nitrogen fixation compared to free-living conditions [see Figure 3(D)]. This finding supports the intuitive idea that coherent activity occurs inside the metabolic modules reported in Figure 3 (B). However, converse to this global trend, we observed a few metabolites with an opposite behavior [see the black lines in Figure 3 (D)]. These components are potentially central metabolites with a specific regulatory control that is perhaps required for transforming the functional background during bacterial nitrogen fixation, a hypothesis to be evaluated in future studies. A list of metabolites integrating each of the structural modules, those metabolites that were experimentally detected by high-throughput technology, the ratio obtained under the two physiological conditions, and their functional classifications according to the KEGG database are depicted in Dataset S2 in the supporting information.

### Metabolic flux activity and functional modules during bacterial nitrogen fixation

The metabolic profile in Figure 1 represents valuable data for surveying how metabolites are organized during bacterial nitrogen fixation on a network scale. In particular, modular organization in biological systems has been suggested as a common property in biological networks at different biological levels [4], [5]. Here, as discussed above, we have supplied evidence that structural modules for the metabolic reconstruction of *R. etli* can be considered a potential organizing principle by which the bacterium orchestrates its physiological response during nitrogen fixation (see Figure 3). However, these modules were identified through a static description, in which physiological information associated with a specific environment was completely absent. In order to study how the topological modules described in Figure 3 can work together to support an optimal phenotype for *R.etli*, we applied FBA to simulate the metabolic flux activity during bacterial nitrogen fixation. The aims in this section are 2-fold: 1) identification of the metabolic flux profile that acts during bacterial nitrogen fixation and to evaluate to what degree the components of the structural modules participate in supporting maximal nitrogen fixation, and 2) evaluation of the extent to which the metabolic array required for reaching a maximal phenotype for bacterial nitrogen fixation is robust under perturbations of the physiological conditions.

As we described above, data-driven reconstruction of the OF represents a significant contribution to simultaneously improving the predictive scope of constraint-based modeling and unveiling the structural organization for cell metabolism. Hence, we proceeded to apply FBA to the metabolic reconstruction for *R.etli* to obtain the flux metabolic distribution that maximizes the metabolome-driven OF described above. The solution was found by solving the linear optimization problem, which was subject to thermodynamic and enzymatic constraints, over the entire set of biochemical reactions in the metabolic reconstruction (see the Materials and Methods section and Dataset S3 in the supporting information). Having applied FBA, we identified those reactions required for optimizing nitrogen fixation and, with them, reconstructed a subnetwork involving only their corresponding substrates and products (see Materials and Methods).

**Figure 4:**
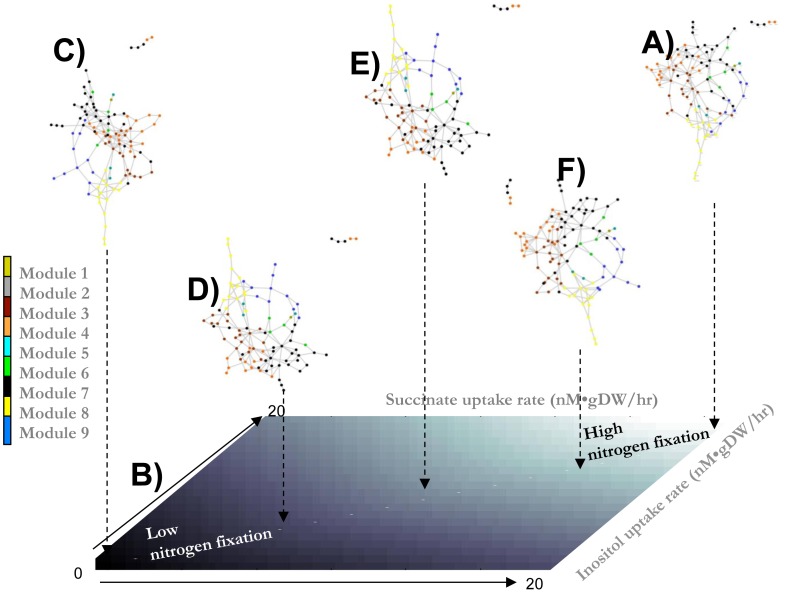
Robust modular composition at different uptake rates of carbon sources. Figure (A) represents the fraction of metabolites required to optimize bacterial nitrogen fixation induced from FBA. Region (B) denotes the phenotype phase plane when inositol and succinate uptake rates were varied from 0 to 20 nM/gDW/hr. The black and white regions indicate low and high nitrogen fixation activities, respectively. Figures (C–F) denote the topological structures for metabolism that ensure optimal nitrogen fixation in the bacteria under different conditions of succinate and inositol uptake rates. Overall, we selected 20 different physiological conditions, corresponding to the diagonal line in the phenotype phase plane that is shown in (B).

To dissect the functional participation of the metabolites integrating this subnetwork, we took into account the modular classification previously defined in Figure 3(B) and Dataset S2 in the supporting information. As expected, we identified the metabolites that potentially participate in support of bacterial nitrogen fixation, their biological roles, and their distributions along the modules (see Figure 4 (A) and the Materials and Methods section).The metabolic properties inferred from this subnetwork were such that 47.96% (47 of 98) of the metabolites predicted by constraint-based modeling were experimentally detected inside the bacteroids for *R.etli*. Even though this percentage of alignment with computational modeling is relatively low, this finding represents a significant advance towards a more realistic method for the study of the metabolic activity of bacterial nitrogen fixation. As far as our knowledge extends, this metabolome study is the first performed for *Rhizobiaceas* in such a way that it defines a benchmark point for future improvements. According to the functional classification described in Figure 3(A) and in Dataset S2 at supporting information, our *in silico* analysis suggested that nitrogen fixation requires a variety of metabolites, mostly peptides and lipids, belonging to diverse structural modules. Converse to the idea that all the metabolites in a specific module participate during this biological process, we found that a metabolic heterogeneity—in terms of both the number of components and module classification—is required for optimizing bacterial nitrogen fixation in *R.etli*.

Notably, the subnetwork depicted in Figure 4(A) shows a few metabolites that participate in the synthesis of nucleic acids (dark green dots). These findings are in qualitative agreement with the fact that *R. etli* bacteroids do not grow during symbiotic nitrogen fixation with *P. vulgaris* (bean plant). In order to distinguish the modules obtained from pure topological criteria from modules specific for a physiological condition, here after we denote these latter as functional modules. At this stage, a question that immediately emerges is to what extent the functional modules depicted in Figure 4 (A) are robust under external environmental perturbations, and also whether this network array can be used as a fingerprint to characterize a functional phenotype in bacterial nitrogen fixation.

### Robustness of the metabolic profile underlying functional states

**Figure 5:**
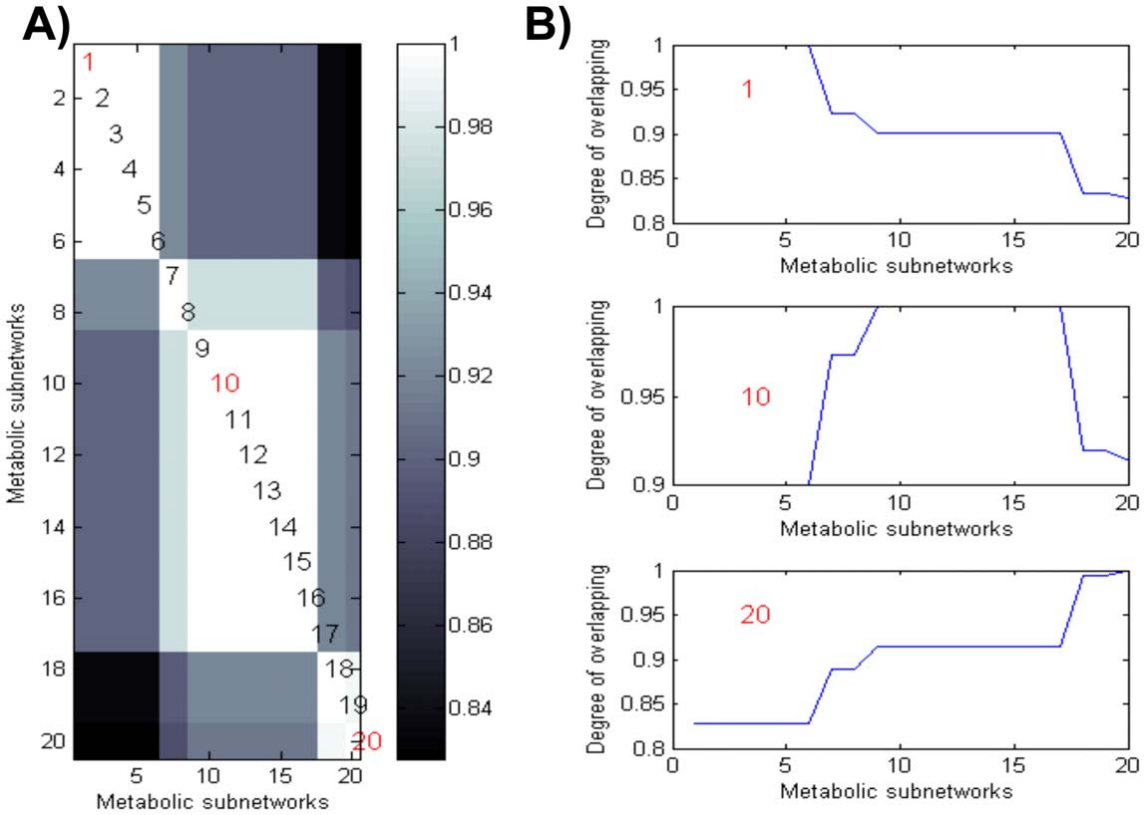
Matrix of overlapping networks. Figure (A) graphically depicts the degree of similitude between the two subnetworks represented in Figure 4 (C–G). In this matrix, an entry located at row *i* and column *j* indicate the comparative analysis between any pair of metabolic subnetworks, *i* = 1.20 and *j* = 1.20. For each entry, white and black indicate the highest and smallest degree of similitude (overlapping) between two subnetworks, respectively. Intermediary values are represented in gray. The degree of overlapping obtained between the three subnetworks (indicated in red) with the rest of the subgraphs depicted in Figure 4 are shown in (B). As panel (B) shows, most of the subgraphs are similar around its vicinity in the phase plane, and an overlap of 0.83 was obtained for subnetworks obtained with extreme points in fluxes in the phase plane.

For the purpose of evaluating whether the metabolic organization shown in Figure 4(A) can be used as a fingerprint to define the optimal phenotype for bacterial nitrogen fixation in *R. etli*, we explored to what extent this topological structure is robust during changes under external environmental conditions. To this end, we evaluated the robustness of this metabolic array through the systematic reduction of uptake rates of two carbon sources: succinate and inositol. While the plant supplies succinate to the bacteroid as the main carbon source, inositol is an internal metabolite that has been detected at high concentrations inside bacteroids in nodules. As has been experimentally verified, both metabolites are important components in the support of nitrogen fixation in *Rhizobiaceas* [11]. To explore how the availability of inositol and succinate alter the metabolic phenotype shown in Figure 3(A), we constructed a phenotype phase plane over these carbon sources [see Figure 4 (B)]. In agreement with the physiological knowledge for *Rhizobiaceas*, we observed that according to the uptake rate, carbon sources in bacterial metabolism were reduced, and a reduction effect on nitrogen fixation was obtained [see the black region of low nitrogen fixation in Figure 4(B)]. To evaluate the topological changes at different points in the phenotype phase plane, *i.e.*, with different succinate and inositol uptake rates, we selected a subset of 20 points along the metabolic phase plane. As depicted by the diagonal line in Figure 4(B), uptake rates for both carbon sources were selected such that their fluxes simultaneously decreased from 20 to 0 nmol of gDW/hr. Then, for each one of the selected uptake rate conditions, we graphically represented the metabolic subnetwork that resulted from FBA. As described in the Materials and Methods section, these subnetworks were constructed by considering only those metabolites participating in the biochemical reactions required for optimization of the phenotype under defined conditions of succinate and inositol uptake rates [see Figure 4 (C–F)]. To quantify the topological variations that resulted under different external conditions, we defined an overlapping coefficient as the fraction of metabolites that overlap these networks (see the Materials and Methods section). With the purpose of quantifying the differences among all pairs of subnetworks, we constructed an overlapping matrix whose calculated numerical entries indicated the overlapping coefficient for each pair of subnetworks obtained at different succinate and inositol uptake rates. The comparative analysis over the 20 points depicted in Figure 4(B) is shown in Figure 5.This study led us to conclude that the metabolic profile required for optimizing bacterial nitrogen fixation does not change in a significant way for a wide range of uptake rates for either carbon source [see Figure 5 (A–B)]. Hence, our study supplies evidence that, while limited carbon sources reduce the bacterial phenotype, the metabolic organization supporting bacterial nitrogen fixation tends to be robust at different succinate and inositol uptake rates. In other words, even though there was a significant reduction for a phenotype, the topology of the metabolic network did not change in a significant fashion. In light of these results, we concluded that functional nitrogen fixation, at low or high rates, seems to be achieved through a conservative metabolic profile involving those metabolites required for support of optimal nitrogen fixation under specific environmental conditions (see Figure 5). Notably, this property opens the possibility for the use of network topology for characterizing cellular phenotypes through a specific pattern of metabolites and potentially distinguishing functional from dysfunctional states associated with a specific biological system. This latter approach is an avenue to be addressed in the future.

Converse to the naive idea that a reduced phenotype in bacterial nitrogen fixation is the consequence of a broken modularity in metabolism, our study supplies evidence that a reduced efficiency of a phenotype can be mainly a consequence of a decrement in flux activity along the network, but without a significant rupture of the functional modules defined in Figure 4(A). In this contextual scheme, modular organization in metabolism seems to be a necessary condition for supporting not only the maximal but a suboptimal functional phenotype in bacterial nitrogen fixation (see Figure 4).

## Discussion

A description of the integration between high-throughput data and computational modeling is a central issue in systems biology that is required for moving toward a quantitative and predictive analysis of the metabolic activity in microorganisms. This enterprise has relevant implications, not only in solving practical issues in biotechnology but also in supplying schemes that contribute to unraveling the principles and mechanisms that regulate and organize living systems.

In this work, we have explored the relationships between three biological concepts in metabolic networks: structural modularity, biological functionality, and optimal (maximal) phenotype. Here, the relationships among these concepts were studied at a systems level for metabolism in bacterial nitrogen fixation, an important biological process participating in the balance of nitrogen in the biosphere [19]. Hence, by using constraint-based modeling and measuring metabolome data under two physiological conditions, we explored the metabolic organization in *R.etli* bacteroids while they fix nitrogen in symbiotic association with *P. vulgaris* (bean plants).

Unlike our previous study [11], here we present a refined version of the OF used for simulating bacterial nitrogen fixation based on our taking into account metabolome data. When constraint-based modeling was applied in this new context, we concluded that 71% of the metabolic reactions predicted *in silico* were justified by the proteome and microarray data previously stored in the GEO database [see Figure 2(D) and the Materials and Methods section].

On the other hand, based on the metabolic reconstruction for *R.etli*, we identified nine structural modules in which some of the metabolites components fell in one of the following biological roles: nucleic acids, peptides or lipids (see Dataset S2 in supporting information). Furthermore, by considering the metabolome profile of 220 metabolites under two physiological conditions, we supplied evidence that most of the metabolites integrating each one of these modules works in a coordinated fashion by increasing their metabolite concentrations at nitrogen fixation stages [see Figure 3 (D)]. This latter finding supports the idea that metabolites inside a module tend to respond in a coordinated fashion. This systems-level description supplies evidence that the diverse metabolites forming the modules participate to support an optimal phenotype in bacterial nitrogen fixation. Even more fundamentally, we note that the network representation for those arrays of metabolites associated with an optimal metabolic performance is robust under different external conditions [see Figures 4 and 5].

In light of this study's findings, the main contributions can be summed up as follows:1) we have supplied evidence that a robust modular organization at the metabolic level underlies optimal bacterial nitrogen fixation for *R. etli*; 2) we have proposed how these functional modules interact together for supporting bacterial nitrogen fixation; and 3) given the robustness observed for these functional modules when there are physiological changes, we suggest that these can be used as fingerprints to associate the active topological structure in a network with optimal phenotypic behavior. Why the metabolic activity is so robust under environmental changes?, maybe a common explanation can be found in other complex systems, for instance the social organization in factories, where even though the production of cars can change as a consequence of external factors—as low or high demand—the hierarchical and modular organization among employees is still maintained for covering a specific demand in an efficient way.

Finally, even though a variety of avenues should be addressed in future research to enrich this study, we envision that this unified description will contribute to the design of experiments for evaluating the interrelation and the role that these modules have in supporting functional states in bacterial nitrogen fixation. This latter issue is fundamental for uncovering the principles by which the cell organizes its metabolism to support functional states in bacterial nitrogen fixation.

## Materials and Methods

### Bacterial strains, growth conditions, and plant experiments

The bacterial strain used was *R. etli* CFN42 wild type. Culture media and growth conditions for *R. etli* and plant experiments were performed as previously described by our group in reference [11].

### Constraint-based modeling

Metabolic flux distribution supporting nitrogen fixation in *R.etli* was predicted *in silico* by using constraint-based modeling [19]. Briefly, simulations were carried out by defining a mathematical function, called the OF, for computationally mimicking the metabolism of bacterial nitrogen fixation and identifying the flux distribution that maximizes it at a steady-state behavior. The OF, *Z^Fix^*, is composed of some key compounds, which are classified in two groups: 1) metabolites essential for sustaining nitrogen fixation, and 2) metabolites that are imported or exported to the bacteroid and establish the symbiotic relationship between *R. etli* and the plant (these are denoted by the index [e]). Thus, we write the OF as follows:
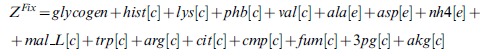
where glycogen, histidine, lysine, polyhydroxybutyrate, valine, alanine, aspartate, and ammonium are denoted as *glycogen*, *hist*[c], *lys*, *phb*[c], *val[c]*, *ala*[e], *asp*[e], and *nh4*[e], respectively. Similarly, *mal*, *trp*, *arg*, *cit*, *cmp*, *fum*, *3pg*, and *akg* denote malate, tryptophan, arginine, citrate, CMP, fumarate, 3-phospho-d-glycerate, and 2-oxoglutarate, respectively. All these metabolites are required to support an effective symbiotic nitrogen fixation, and their spatial location in the cytoplasm is indicated by the index [c]. For the purpose of obtaining a computational profile of metabolic fluxes, we assumed that the metabolic state of the bacteroid during nitrogen fixation is one that optimizes the OF, *Z^Fix^*. This problem was mathematically solved as a linear optimization algorithm subject to enzymatic and thermodynamic constraints, *i.e.*,
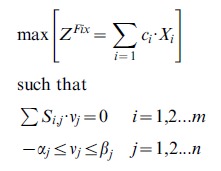
where *S_i,j_* represents the stoichiometric coefficient of metabolite *i* participating in the *j*th reaction. Thermodynamic and enzymatic constraints in each metabolic reaction were characterized through the parameters *α_j_* and *β_j_* (for a detailed numerical description of these parameters, see the lower and upper bounds in Dataset S3 in supporting information). For the sake of simplicity, all the coefficients (*c_i_*) were chosen as a unit along all the analyses. Linear optimization programming was carried out using the Tomlab optimization package from Matlab.

### Metabolome data and inference of the OF for bacterial nitrogen fixation: statistical test of metabolite relative expression levels

Metabolome data supply valuable information to survey the metabolic phenotype associated with a biological process, and in turn, elucidate the components integrating the OF used in the constraint-based modeling. With the purpose of carrying out FBA in the metabolic reconstruction for *R.etli*, a more realistic OF for simulating bacterial nitrogen fixation was obtained by identifying those metabolites with a statistically significant change during bacteroid activity compared to levels under free-living conditions. To this end, we calculated the corresponding intensity log-ratio for each metabolite between nitrogen fixation and free-living conditions. Taking into account the three experimental replicates obtained for each physiological condition, a one-side statistical *t*-test was applied to determine those metabolites that significantly increased their relative quantity between the two physiological conditions (see Figure 1). Those metabolites with a *p-value* lower than 0.01 and with a ratio higher than 2 during nitrogen fixation were selected as potential candidates to be included in the OF, see region I in Figure 2(A). Finally, these sets of metabolites were separately included in the OF and were accepted if their effect on nitrogen fixation activity was not reduced to one-third of the corresponding previous OF.

### Transcriptome and proteome data

To assess the predictive scope of the computational model, we downloaded transcriptome and proteome data so that we could integrate a set of genes whose presence and upregulation suggested an important role for sustaining nitrogen fixation in *R.etli*. Earlier characterizations of nodules formed after 18 days of nodulation with root plants under both experimental conditions were carried out by our group and previously stored in public databases. The complete dataset of the transcriptome analysis is freely available at GEO (http://www.ncbi.nlm.nih.gov/geo), with accession numbers GPL10081 for the *R.etli* platform and GSE21638 for data on free-living and symbiotic forms. In addition, to enrich the set of genes required for model assessment, proteome data obtained for *R.etli* bacteroids were downloaded from the ProteomeCommons.org Tranche by using the following hash:BY/eCcVjwTWN1+m+2ArvJ0QVnesGx5Ekgd4wUOASACfm/ueNl7YI3iLf4xz0lnGsepV5LkpMWOQOrZtjYExlNpQkIBcAAAAAAAABjA =  = .

### Measurement of ionic metabolites by using the CE-MS system

Bacteria were grown in minimal medium and were harvested at the exponential phase. Free-living cells were collected by centrifugation and washed once with double-distilled water, and immediately 2 ml of methanol and the internal standards were added and the mixture was treated in an ultrasonic bath for 30 s. A 1.6-ml cell suspension was transferred to centrifuge tubes,1.6 ml of CHCl_3_ plus 640 µl of milliQ water was added, and the mixture was vortexed and then centrifuged at 2,300×*g*at 4°C for 5 min. A 1.5-ml aliquot of the aqueous layer was filtered through a Millipore 5-kDa cutoff filter. Then, the sample was fully dried by using a centrifugal evaporator. The bacteroid metabolome extraction followed the same method, except by day 18 after inoculation with root plant, bacteroids were extracted in a self-Percoll gradient in accordance with methods previously described [21]. Measurements of extracted metabolites were performed by using CE coupled with electrospray ionization–time-of-flight analysis and MS with electrophoresis buffer (solution ID H3302-1021; Human Metabolome Technologies Inc., Tsuruoka, Japan).

### Definition of the consistency coefficient

To assess agreement between *in silico* predictions and interpretations of high-throughput data, we defined a consistency coefficient that quantified the fraction of genes predicted to be upregulated *in silico* and simultaneously detected or induced by proteome or transcriptome technologies (*η_Gene_*). Simultaneously, we defined a consistency coefficient that quantified the fraction of active enzymes that were predicted by constraint-based modeling and identified by high-throughput technology (*η_Enzyme_*). To proceed with this evaluation, *E ^j^_kegg_*(*G ^j^_kegg_*) was denoted as the set of enzymes (genes) that conformed to the *j*-esime metabolic pathways in the KEGG database, with *j* ranging from 1 to 22. Similarly, the set of enzymes (genes) that integrated the *i*-esime metabolic pathway in the reconstruction and the set of enzymes that were detected by high-throughput data were denoted *E ^j^_Rec_*(*G ^j^_Rec_*)and *E ^j^_HT_*(*G ^j^_HT_*), respectively. Finally, the set of enzymes obtained from constraint-based modeling were denoted *E ^j^_iModel_* and*G ^j^_iModel_*. In order to evaluate and create a proper frame of comparison between *in silico* predictions and high-throughput data, we defined the consistency coefficient as the fraction of enzymes (genes) that were actively predicted *in silico* and were identified by high-throughput technology as follows:
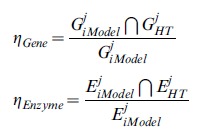
This ratio ranged from 0 to 1 and constituted our central parameter to assess and quantify the agreement between the constraint-based modeling and high-throughput data. To assess the output from FBA, we selected 22 metabolic pathways and evaluated the degree of coherence between the flux activity predicted *in silico* with data for the proteome and transcriptome previously reported for *R.etli* during nitrogen fixation (see reference [11]).

### Network representation of the metabolic network

Topological analysis was accomplished by representing the set of biochemical reactions in the metabolic reconstruction as an undirected network. In this network, nodes represent the metabolites and edges indicate their participation in a specific metabolic reaction [4]. Thus, we linked the metabolites in the reactants to all the products in the biochemical reaction, and this procedure was repeated for all the reactions included in the metabolic reconstruction, to finally obtain the results shown in Figure 3(B). In the case of the FBA, the subnetworks depicted in Figure 4(A), (C–F) were obtained when we applied these rules only to those metabolic reactions for which the flux obtained from FBA was different from 0. In order to analyze those metabolites with biological roles, the following metabolites were excluded from the entire network representation: pi[e], ala-L[e], accoa[e], glu-L[e], pi[c], fdp[c], nh4[e], nh3[c], nh3[e], h[e], ppi[c], o2[c], o2[e], nadph[c], nadp[c], nadh[c], nad[c], n2[c], n2[e], h2o2[c], h2o[c], h2o[e], h[c], fdred[c], fdox[c], fadh2[c], co2[c], co2[e], nh4[c], and fad[c]. A detailed list of the metabolic reconstruction *iOR450* used in this work, including the metabolic reactions and metabolite abbreviations, is provided in Dataset S3 in supporting information.

### Module identifications

Modular composition along the entire metabolic reconstruction was obtained by a clustering analysis applied on a matrix whose entries represented the inverse squares of minimal path lengths for pairs. Specifically, the clustering analysis was performed by calculating the shortest path length between every pair of genes (*i.e*.,*d_ij_* is the shortest path length between gene *i* and gene *j*). Next, we calculated the association function, defined as 1/*dij* ^2^, for all pairs of metabolites along the metabolic reconstruction. This parameter gives a measure of the closeness among genes, amplifying the parameter for pairs of metabolites with low path lengths and minimizing pairs of metabolites located at remote distances. With the purpose of identifying topological modules along the network, these sets of parameters were used as input to perform a hierarchical clustering [3], [4], [5], [22], see Figure 2(A). A detailed description of the modules identified by this algorithm and the corresponding biological roles for some of the components are shown in Dataset S2. The clustering analysis was performed using a hierarchical agglomerative average-linkage clustering algorithm, considering Kendall'sτ value as the similarity metric.

### Overlapping coefficient among modules

In order to compare the similarities and differences that emerged from the functional modules obtained along the phase plane region depicted in Figure 4, we define the following coefficient:
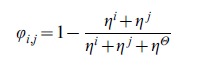
where *η^i^* and *η^j^* represent the number of metabolites that appeared in only module *i* or *j*, respectively, and 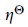 indicates the number of metabolites in common between modules *i* and *j*. Clearly, this coefficient ranges from 1 to 0,depending on the complete or null analogy between the *i* and *j* functional subnetworks.

## Supporting Information

Dataset S1 **Metabolome data.** This file contains the metabolome data set obtained experimentally through CE-MS. The table shows those metabolites whose concentrations were differentially up-regulated and down-regulated during bacterial nitrogen fixation respect to free-living conditions. In addition, we report those metabolites experimentally detected only in one of the physiological states analyzed.
(XLSX)

Dataset S2 **Metabolites integrating each structural module.** This table contains information of the metabolites forming each one of the nine structural modules identified by the topological criteria used in the main text.
(XLSX)

Dataset S3 **Genome-scale metabolic reconstruction for** ***Rhizobium etli*** **(** ***iOR450*** **).** This file contains the entire set of biochemical reactions included in the metabolic reconstruction for *R. etli*. Overall, the metabolic reconstruction of *R. etli* is an integrated network of 402 reactions involving the participation of 450 genes and 377 metabolites.
(XLSX)
